# Moyamoya Disease With Initial Ischemic or Hemorrhagic Attack Shows Different Brain Structural and Functional Features: A Pilot Study

**DOI:** 10.3389/fneur.2022.871421

**Published:** 2022-05-13

**Authors:** Junwen Hu, Yin Li, Yun Tong, Zhaoqing Li, Jingyin Chen, Yang Cao, Yifan Zhang, Duo Xu, Leilei Zheng, Ruiliang Bai, Lin Wang

**Affiliations:** ^1^Department of Neurosurgery, The Second Affiliated Hospital, Zhejiang University School of Medicine, Hangzhou, China; ^2^Zhejiang University School of Medicine, Hangzhou, China; ^3^Key Laboratory of Biomedical Engineering of Education Ministry, College of Biomedical Engineering and Instrument Science, Zhejiang University, Hangzhou, China; ^4^Department of Radiology, The Second Affiliated Hospital, Zhejiang University School of Medicine, Hangzhou, China; ^5^Department of Psychiatry, The Second Affiliated Hospital, Zhejiang University School of Medicine, Hangzhou, China; ^6^Department of Physical Medicine and Rehabilitation, The Affiliated Sir Run Run Shaw Hospital and Interdisciplinary Institute of Neuroscience and Technology, Zhejiang University School of Medicine, Hangzhou, China

**Keywords:** moyamoya disease, multimodality MRI, cortical thickness, fractional anisotropy, arcuate fasciculus

## Abstract

**Objective:**

Cerebral ischemia and intracranial hemorrhage are the two main phenotypes of moyamoya disease (MMD). However, the pathophysiological processes of these two MMD phenotypes are still largely unknown. Here, we aimed to use multimodal neuroimaging techniques to explore the brain structural and functional differences between the two MMD subtypes.

**Methods:**

We included 12 patients with ischemic MMD, 10 patients with hemorrhagic MMD, and 10 healthy controls (HCs). Each patient underwent MRI scans and cognitive assessment. The cortical thickness of two MMD subtypes and HC group were compared. Arterial spin labeling (ASL) and diffusion tensor imaging (DTI) were used to inspect the cerebral blood flow (CBF) of cortical regions and the integrity of related white matter fibers, respectively. Correlation analyses were then performed among the MRI metrics and cognitive function scores.

**Results:**

We found that only the cortical thickness in the right middle temporal gyrus (MTG) of hemorrhagic MMD was significantly greater than both ischemic MMD and HC (*p* < 0.05). In addition, the right MTG showed higher ASL-CBF, and its associated fiber tract (arcuate fasciculus, AF) exhibited higher fractional anisotropy (FA) values in hemorrhagic MMD. Furthermore, the cortical thickness of the right MTG was positively correlated with its ASL-CBF values (*r* = 0.37, *p* = 0.046) and the FA values of right AF (*r* = 0.67, *p* < 0.001). At last, the FA values of right AF were found to be significantly correlated with cognitive performances within patients with MMD.

**Conclusions:**

Hemorrhagic MMD shows increased cortical thickness on the right MTG in comparison with ischemic MMD and HCs. The increased cortical thickness is associated with the higher CBF values and the increased integrity of the right AF. These findings are important to understand the clinical symptoms and pathophysiology of MMD and further applied to clinical practice.

## Introduction

Moyamoya disease (MMD) is a rare cerebrovascular disease characterized by the chronic progressive steno-occlusive changes of terminal internal carotid arteries and a hazy vascular network of basal collaterals (1). Cerebral ischemia and intracranial hemorrhage are the two main phenotypes of MMD, with ischemic MMD being slightly more frequent than hemorrhagic MMD (2). The causes of both the phenotypes of MMD are different, the former is attributed to a vessel occlusion that leads to the hypoperfusion of brain (3), while the latter is mainly a result of the rupture of fragile moyamoya vessels or cerebral aneurysms (3). Due to the differences in etiological factors, there is a high likelihood that hemodynamic impairment may have different effects on the brain structure and function between the two phenotypes. However, it remains unknown.

Multimodal imaging techniques, such as structural imaging, diffusion tensor imaging (DTI), and arterial spin labeling (ASL) technique, provide a multifaceted approach to explore the structural and functional changes in the brain. Such knowledge could help us achieve a better diagnosis and an improved prognosis of MMD. Cortical thickness is one of the essential parameters of brain structure, which could provide us further information about the pathogenesis and neuropathology of the disease (4, 5). Previous studies reported that patients with MMD have reduced gray matter volume in the anterior portion of the cingulum and some parts of the frontal and occipital gyrus (6, 7). In addition, a few studies have documented cortical thickness changes in patients with MMD compared with healthy controls (HCs) (8, 9). DTI has become one of the most popular magnetic resonance imaging (MRI) techniques for characterizing the white matter microstructure features and detecting whether the neuron fibers and tissue are damaged on the micron scale (10). Fractional anisotropy (FA) is the most frequently used metric for characterizing the tissue microstructure. Microstructure changes, such as those reflected on these metrics have been correlated to the clinical status of patients with MMD, especially in cognitive measures (11). ASL is a novel technique that could quantitatively measure the cerebral blood flow (CBF) in a non-invasive way by magnetically labeling the blood water protons (12). It has been verified in various diseases and has great application in neurovascular diseases, such as those similar to MMD (13). However, most of these studies were focused on patients with ischemic MMD or MMD patients without separating subtypes, and little is known about the difference of structural and functional brain features between hemorrhagic and ischemic MMD.

Here, we aimed to investigate the brain structural and functional differences between patients with ischemic and hemorrhagic MMD by the virtue of multimodal imaging. We selected patients with ischemic and hemorrhagic MMD without apparent lesions in the conventional MRI to avoid potential bias caused by lesions in the comparison of brain images (e.g., registration, segmentation, and parcellation). We first explored the cortical thickness of these two MMD subtypes in comparison with the HC group, respectively, in FreeSurfer *via* a general linear model (GLM). Then, the blood flows of the selected cortical regions were studied with the CBF derived from ASL. In addition, the integrity of white matter tracts associated with these selected cortical regions was inspected by the computation of fibers' FA values. At last, correlation analyses were performed among these MRI metrics and MMD cognitive function scores to learn the underlying relationships.

## Methods

### Participants

We performed a prospective, observational, and single-center study. The study was conducted in accordance with the principles of the Declaration of Helsinki and was approved by the Human Research Ethics Committee of Second Affiliated Hospital of Zhejiang University (ID: 2020-064). Informed consent was obtained from each subject. A cohort of 56 patients with MMD was included in this study from July 2020 to May 2021. All patient diagnoses of MMD were based on the digital subtraction angiography (DSA). The diagnostic criteria were in line with the “Consensus of Chinese Experts on the Diagnosis and Treatment of Moyamoya Disease and Moyamoya Syndrome (2017)” (14). Patients who were right-handed and manifested as ischemia or hemorrhage at the initial attack were included, none of the patients were in the acute stage of attacks (with a time to onset of more than 3 months). Exclusion criteria were as follows: incomplete MRI data, revascularization surgery before the study, cortical lesions on T1-weighted images (T1WIs) larger than 3 mm. All imaging data were reviewed by two experienced clinicians (Dr. Xu and Dr. Wang).

In addition, another 10 healthy participants with gender, age, and education matched with the MMD group were recruited as the control group. Inclusion criteria for the control group were no diagnosed brain lesions and no history of neurologic or systematic diseases.

### Cognitive Assessment

All patients diagnosed with MMD were assessed for cognitive function by the same psychiatrist (Dr. Zheng) in our hospital before the MRI scan. The cognitive assessment package included the Mini-Mental State Examination (MMSE), the Montreal Cognitive Assessment (MoCA), and the Trail Making Test parts A and B (TMT-A/-B) which shows the visuoperceptual abilities, processing speed, working memory, and other cognitive functions (15). The points for working memory (range 0–5) were extracted from one of the cognitive domains in the MMSE (16). The total times (in seconds) for the TMT-A and TMT-B were used to represent the direct scores (15). TMT B-A means the subtraction of two values.

### MRI Acquisition

The study was completed on a 3T MR scanner (Discovery MR750, GE) with an 8-channel head coil. The entire protocol included T1WIs, T2-weighted fluid attenuated inversion recovery (T2-FLAIR), pseudo-continuous arterial spin labeling (PCASL), and DTI. The total scan time for each subject was about 30 min. Earplugs and spongy padding were used to reduce the noise and head motion.

T1-weighted images were collected through 3D fast spoiled gradient-echo (3D-FSPGR): repetition time (TR) = 7.4 ms, echo time (TE) = 3.1 ms, flip angle = 8°, field of view (FOV) = 256 mm × 256 mm, slices = 170, and voxel size = 0.8 mm × 0.8 mm × 1.0 mm. T2-FLAIR were acquired with following parameters: TR = 8,400 ms, TE = 145.3 ms, flip angle = 90°, FOV = 512 mm × 512 mm, slices = 40, and voxel size = 0.39 mm × 0.39 mm × 4.0 mm.

The diffusion-weighted images were acquired using 60 motion-probing gradient directions with the *b*-values of 2,000 s/mm^2^ and two repetitions on *b* = 0 s/mm^2^, TR = 9,000 ms, TE = 92.7 ms, flip angle = 90°, FOV = 256 mm × 256 mm, slices = 68, and voxel size = 1.0 mm × 1.0 mm × 2.0 mm. To correct the image distortion, we also obtained reverse phase-coded images.

Pseudo-continuous ASL images without vessel suppression used the following parameters: TR = 4,825 ms, TE = 10.6 ms, flip angle = 111°, FOV = 128 mm × 128 mm, slices = 34, voxel size = 1.56 mm × 1.56 mm × 4.0 mm, and labeling time = 1.5 s. We used a post-labeling delay (PLD) time of 1,500 ms.

### Image Processing

The cortical segmentation of structural images was performed by FreeSurfer *v*6.0 (http://surfer.nmr.mgh.harvard.edu) with the “recon-all” pipeline to automatically perform 31 processing steps for each subject and subsequently obtain cortical thickness, the technical details of these procedures were described in prior publications (17). After preprocessing, a group analysis between the two MMD subtypes and the HC group was performed to detect significant differences in cortical thickness through a general linear model (GLM) by designing the FreeSurfer Group Descriptor (FSGD) file and creating a contrast to remove the effects of gender, age, and education. The cortical thickness maps of bilateral hemispheres were separately mapped onto the FreeSurfer “faverage” surface and smoothed using a Gaussian kernel with a full width at half maximum (FWHM) of 10 mm. A cluster-wise threshold of *p* < 0.001 with the Bonferroni correction was the criterion to determine statistical significance (18). Brain regions having statistically significant clusters were identified as the region of interest (ROI) for *post-hoc* analysis.

MRtrix3 (https://www.mrtrix.org/) was used to perform fiber tracking. Denoising, Gibbs ringing removal, motion, and distortion corrections were performed as the part of preprocessing (19). In addition, bias field correction was processed to eliminate low-frequency intensity fluctuations across the image. After preprocessing, the diffusion tensor could be calculated and then the FA metrics could be obtained. The whole-brain fiber tractography was performed in the individual space of each patient, which is defined as the cutoff of FA values <0.2, angle 45°, minimum length 20 mm, and maximum length 200 mm. We selected 10 million streamlines using a fiber orientation distribution (FOD)-based algorithm and subsequently filtered to one million streamlines using the spherical-deconvolution informed filtering of tractograms (SIFT) algorithm to reduce tractography biases (19). The mean FA values of the white matter tract were obtained by overlying the tract template (HCP1065 tractography atlas, http://brain.labsolver.org/diffusion-mri-templates/tractography) (20), which was registered to each subject using the Advanced Normalization Tools (ANTs) (http://stnava.github.io/ANTs/) through default parameters, on the whole-brain fiber tractography map.

The quantitative ASL-CBF map using 1,500 ms PLD was generated on an Advantage Windows Workstation using FuncTool software attached to the scanner (21). The ASL-CBF images of each subject were normalized to MNI152 space using the ANTs through default parameters. The normalized ASL-CBF images were overlaid with the Anatomical Automatic Labeling (AAL) template with MRIcron (https://www.nitrc.org/projects/mricron/, *v*1.0.20190902), and the mean ASL-CBF value of each ROI was extracted. Image processing procedures were reviewed by another researcher with MRI expertise (PhD. Li).

### Statistical Analysis

Statistical evaluations were performed using GraphPad Prism *v*8.0 (GraphPad Software, San Diego, CA, USA). The non-parametric, unpaired *t*-tests were performed among two groups, and the ANOVA tests with Tamhane's T2 correction were performed to compare the cortical thickness through the *post-hoc* analysis, ASL-CBF values, and FA values of white matter tracts among three groups. Spearman's correlation tests were used to evaluate the relationships between paired datasets. A value of *p* < 0.05 was considered statistically significant.

## Results

### Clinical Characteristics

In this study, a total of 56 patients with MMD were recruited, 34 cases were excluded based on the exclusion criteria, in which five cases had incomplete MRI data, five cases had previous revascularization surgery, and 24 cases had cortical lesions larger than 3 mm. Finally, a total of 22 patients with MMD were included, 12 with ischemia and 10 with hemorrhage, with an average age of 46.8 ± 8.9 years and a gender ratio of 3:19 (men to women). The demographic and clinical characteristics of 22 patients with MMD are demonstrated (Supplementary Table). Besides, there was no statistical difference in age, gender, and education between the MMD and HC groups (Table 1), nor between the two types of MMD and HCs (Table 2). Hemorrhagic MMD had significantly better cognitive functions in working memory than ischemic MMD, but there were no statistical differences in MMSE, MoCA, and TMT-A/B (Table 2).

**Table 1:** Baseline characteristics between MMD and HCs.

	**MMD (*n* = 22)**	**HC (*n* = 10)**	***p* value**
Age, mean ± SD (y)	46.8 ± 8.9	49.7 ± 5.4	0.353
Gender (men/women)	3/19	2/8	0.506
Education (y)	8.7 ± 2.9	9.0 ± 3.7	0.835

*MMD, moyamoya disease; HCs, healthy controls*.

**Table 2:** Baseline characteristics between two types of MMD and HCs.

	**Ischemic MMD (*n* = 12)**	**Hemorrhagic MMD (*n* = 10)**	**HC (*n* = 10)**	***p***
Age, mean ± SD (y)	47.8 ± 9.6	45.7 ± 8.4	49.7 ± 5.4	0.551^&^
Gender (men/women)	2/10	1/9	2/8	0.821^†^
Education (y)	8.8 ± 3.3	8.7 ± 2.7	9.0 ± 3.7	0.978^&^
Mainly effected in the right hemisphere	1/8 (12.5%)	6/9 (66.7%)	/	0.024^*^
MMSE (mean ±*SD*)	24.44 ± 4.59	25.30 ± 3.71	/	0.674^#^
MoCA	20.22 ± 7.26	20.20 ± 6.13	/	0.920^#^
Working memory	2.50 ± 1.60	4.22 ± 0.83	/	0.021^#*^
TMT-A (s)	115.3 ± 65.4	84.13 ± 20.21	/	0.637^#^
TMT-B (s)	213.5 ± 114.7	175.4 ± 69.2	/	0.433^#^
TMT B-A (s)	98.17 ± 52.14	91.25 ± 51.06	/	0.729^#^

*There are nine ischemic MMD and 10 hemorrhagic MMD in MMSE, MoCA, and working memory. Besides, six ischemic MMD and eight hemorrhagic MMD in TMT-A, TMT-B, and TMT B-A. MMD, moyamoya disease; HCs, healthy controls; MMSE, Mini–Mental State Examination; MoCA, Montreal Cognitive Assessment; TMT-A/-B, Trail Making Test parts A and B*.

*^†^Chi-square test. There are patients who have no laterality of the hemorrhage or ischemia, or data loss (see Supplementary Table)*.

*^&^ANOVA tests with Tamhane's T2 correction among three groups*.

*^#^Mann–Whitney test between ischemic MMD and hemorrhagic MMD*.

*p <0.05.

### Changes in Cortical Thickness

**Figure 1:**
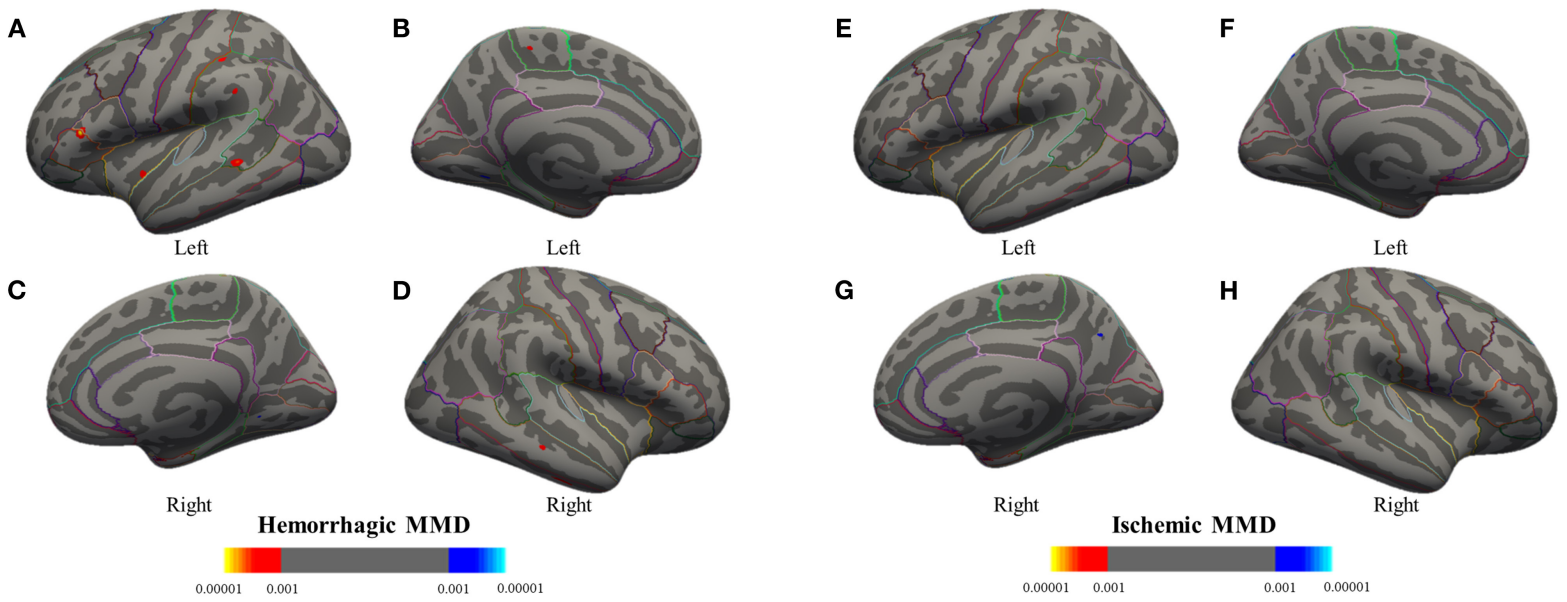
Group analyses between the MMD subtypes and the HC group are performed to detect significant differences in cortical thickness through a general linear model. Results are presented on inflated cortical surfaces. **(A–D)** The lateral and medial surfaces of the bilateral hemisphere of hemorrhagic MMD. **(E–H)** The lateral and medial surfaces of the bilateral hemisphere of ischemic MMD. The red to yellow regions indicate thickened cerebral cortex compared with the HC group (*p* < 0.001, corrected). The blue to azure region represent thinning cerebral cortex (*p* < 0.001, corrected). Hemorrhagic MMD has thickened cortex in the left insula, inferior frontal gyrus, superior temporal gyrus, supramarginal gyrus (SMG), paracentral lobule, and the right middle temporal gyrus while having a thinning cortex in the bilateral lingual gyrus. Ischemic MMD has thinning cortex in the right precuneus and has no thickened cortex. MMD, moyamoya disease; HCs, healthy controls.

As compared with the HC group, GLM revealed that cortical thickness of hemorrhagic MMD increased in several regions, such as the left paracentral lobule, insula, inferior frontal gyrus (IFG), supramarginal gyrus (SMG), and right middle temporal gyrus (MTG). There were no cerebral cortex regions showing significant cortical thickness increases in ischemic MMD with respect to HCs. In addition, the bilateral lingual gyrus (LgG) in hemorrhagic MMD and the right precuneus in ischemic MMD show smaller cortical thickness in comparison with HCs (Figure 1; *p* < 0.001, corrected).

**Figure 2:**
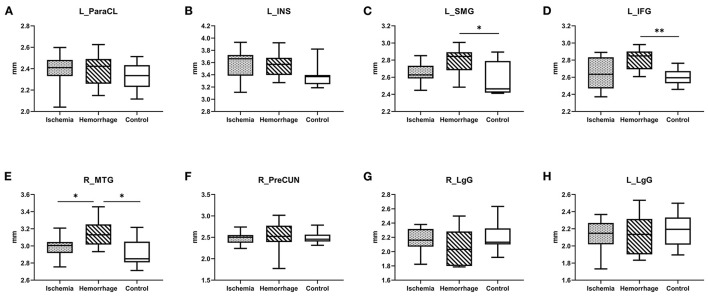
**(A–H)** Comparison of cortical thickness between ischemic MMD, hemorrhagic MMD, and HCs. L means left hemisphere. R means right hemisphere. ParaCL, paracentral lobule; INS, insula; SMG, supramarginal gyrus; IFG, inferior frontal gyrus; MTG, middle temporal gyrus; PreCUN, precuneus; LgG, lingual gyrus. *p < 0.05; **p < 0.01.

A *post-hoc* analysis further confirmed that the cortical thickness in hemorrhagic MMD was significantly increased in the left SMG (*p* < 0.05), IFG (*p* < 0.01), and the right MTG (*p* < 0.05) in comparison with the HC group (Figure 2). In the right MTG, the cortical thickness in hemorrhagic MMD was significantly greater than those in both ischemic MMD and the HC group (*p* < 0.05). Other cortical regions revealed by GLM method do not show significant changes in this *post-hoc* analysis. In the following analysis, only the right MTG was considered as a brain cortical region having significantly different cortical thicknesses between hemorrhagic MMD and HCs and between the two MMD subtypes.

### ASL-CBF Values in the Right MTG

**Figure 3:**
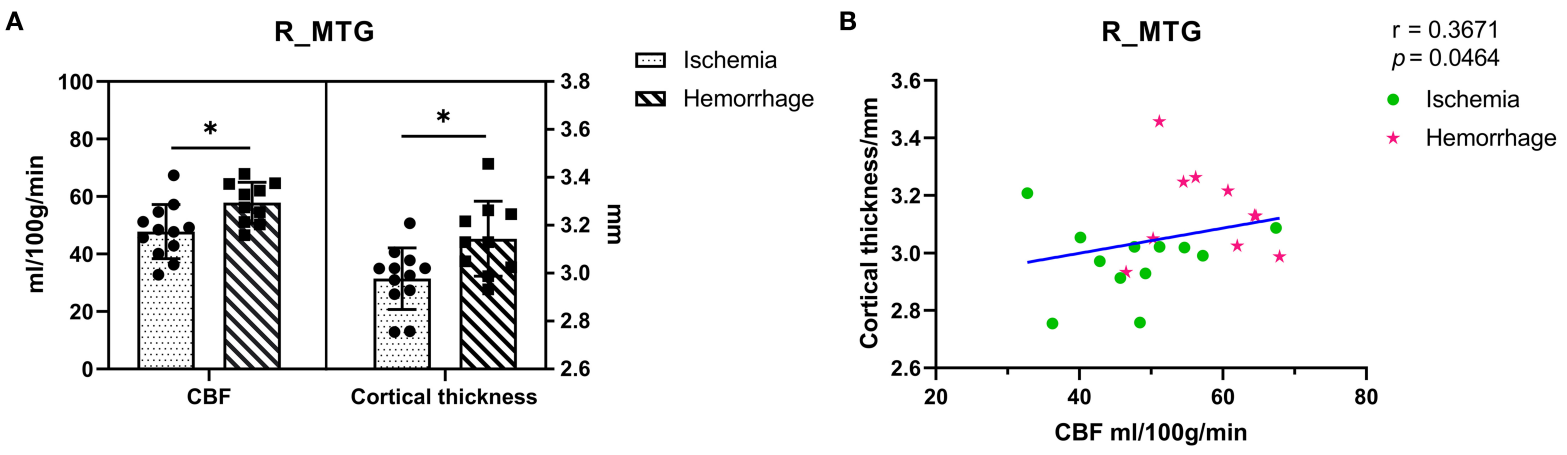
**(A)** Comparison of the ASL-CBF values and cortical thickness between ischemic MMD and hemorrhagic MMD in the right MTG. The left *y*-axis shows the CBF values and the right *y*-axis shows the cortical thickness. **(B)** Correlation between the right MTG and its ASL-CBF within patients with MMD. L means left hemisphere. R means right hemisphere. CBF, cerebral blood flow; MTG, middle temporal gyrus. *p < 0.05.

The ASL-CBF values of hemorrhagic MMD were significantly higher than ischemic MMD in the right MTG (*p* < 0.05) (Figure 3A). In addition, a positive correlation between ASL-CBF values and cortical thickness was seen in the right MTG within patients with MMD (*r* = 0.37, *p* = 0.046) (Figure 3B).

### FA Values in Bilateral Superior Longitudinal Fasciculus and Arcuate Fasciculus

**Figure 4:**
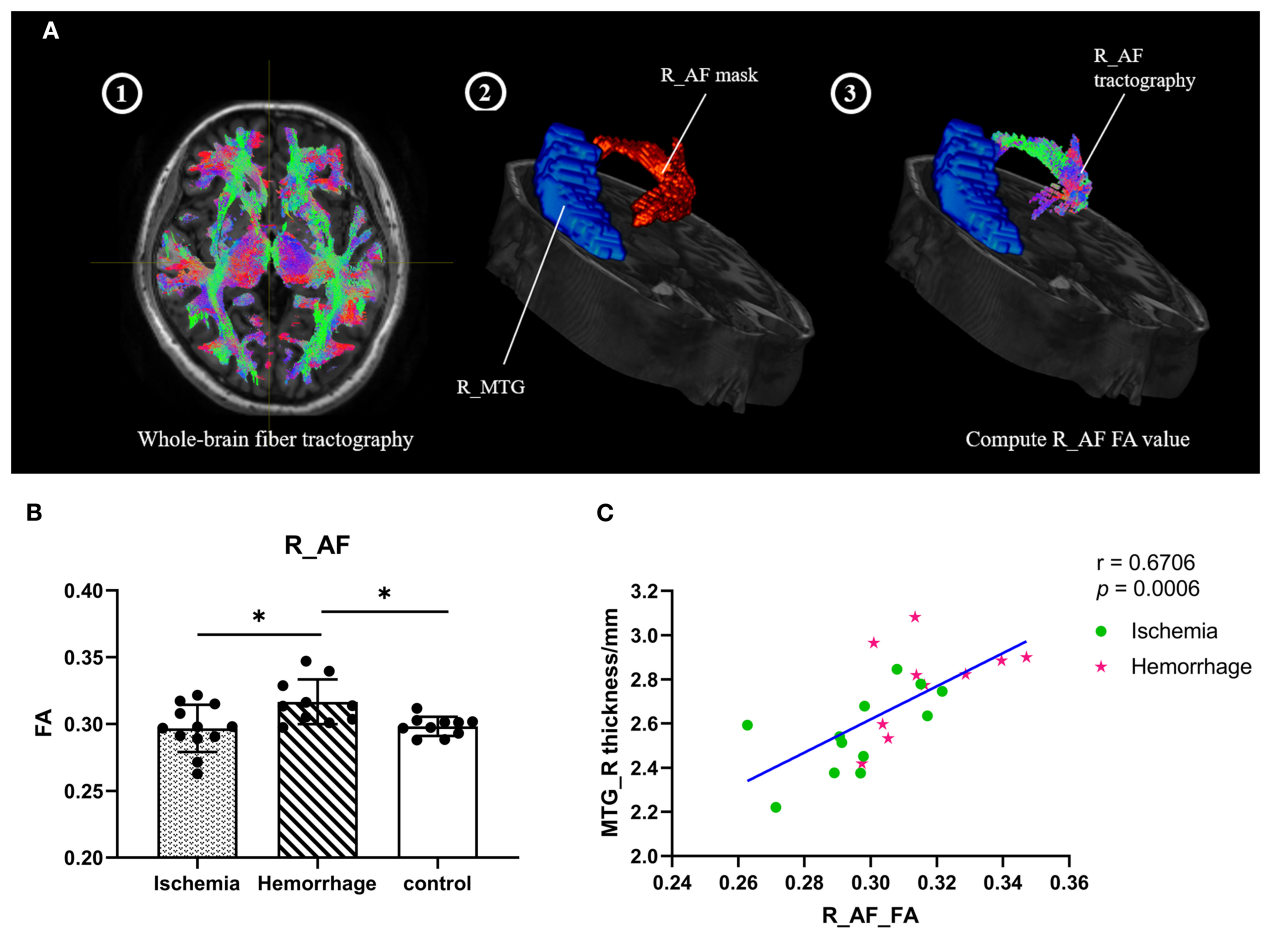
**(A)** Process of computing the FA values of fiber tracts (take the right AF as an example). Step 1: Perform the subject's whole-brain fiber tractography by MRtrix3; Step 2: Overlay the right AF mask; Step 3: Create the right AF tractography and compute the FA values. **(B)** FA values of the right AF between three groups. **(C)** Correlation between the cortical thickness of right MTG and the FA values of right AF within patients with MMD. L means left hemisphere. R means right hemisphere. AF, arcuate fasciculus; MTG, middle temporal gyrus; FA, fractional anisotropy. ^*^ *p* < 0.05.

The superior longitudinal fasciculus (SLF) and arcuate fasciculus (AF) are the two major fiber tracts communicating with the temporal and frontal lobes, especially among those that have projections to the MTG (22, 23). To explore the microstructure changes of these fibers, the mean FA values of fibers were calculated (Figure 4A). Interestingly, hemorrhagic MMD showed higher FA values in the right AF as compared with ischemic MMD and HCs (*p* < 0.05) (Figure 4B), suggesting increased neurite density or myelin thickness in the AF of hemorrhagic MMD (10). No significant changes of the FA values were found in the bilateral SLF and the left AF. In addition, there was a positive correlation between the FA values of the right AF and cortical thickness of the right MTG (*r* = 0.67, *p* < 0.001) (Figure 4C).

### Correlation Between the Right AF and Cognitive Performances

**Figure 5:**
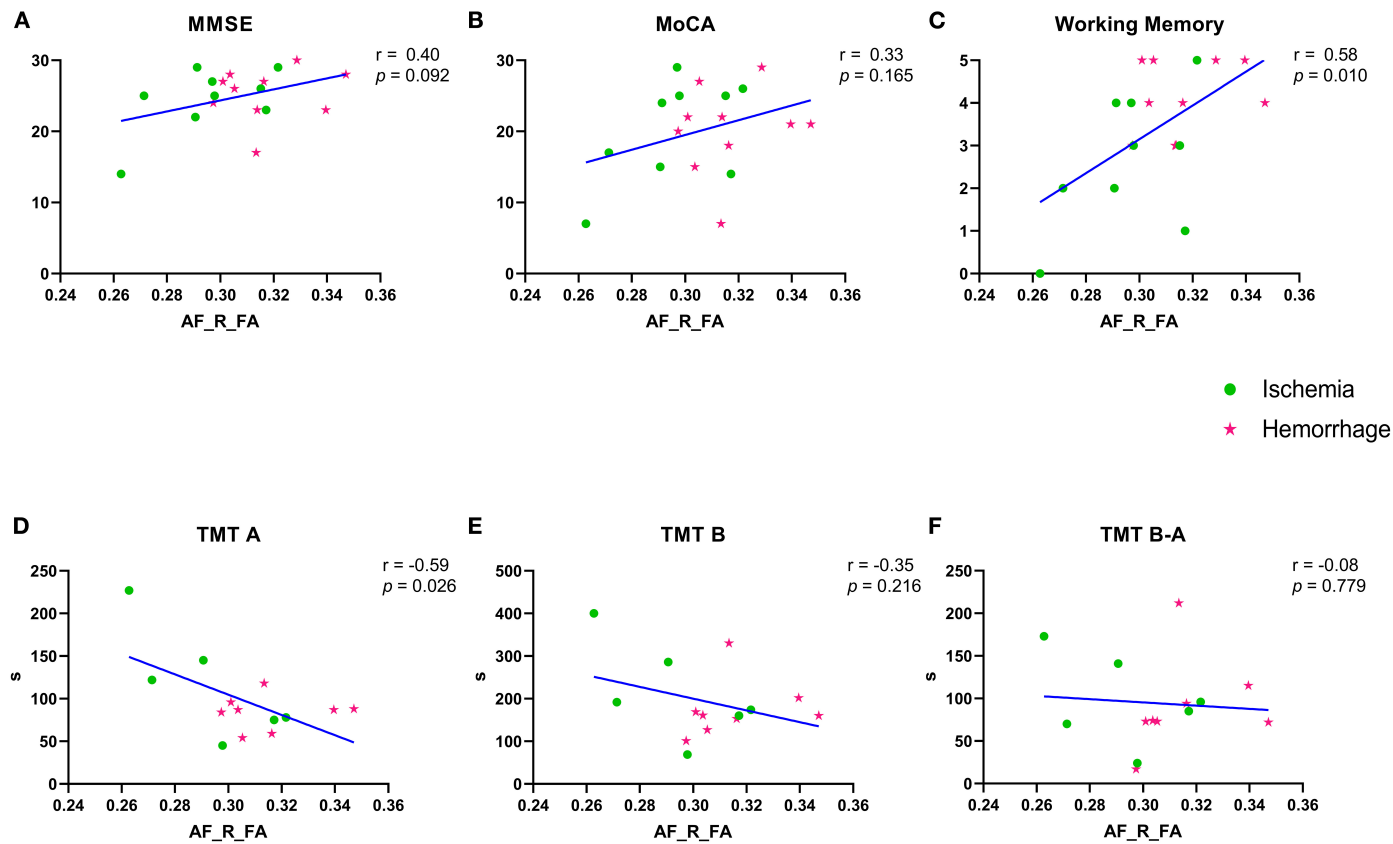
**(A–F)** Correlation between the FA values of right AF and variable cognitive performances. Green spots represent the ischemia group and red stars represent the hemorrhage group. MMSE, Mini–Mental State Examination; MoCA, Montreal Cognitive Assessment; TMT-A/-B, Trail Making Test parts A and B; FA, fractional anisotropy; AF, arcuate fasciculus. R means right. TMT B-A means the subtraction of two values.

Due to data loss in the early collection stage of this study, there were nine patients with ischemic MMD and 10 patients with hemorrhagic MMD with completed MMSE, MoCA, and working memory, which was derived from the MMSE. Besides, six patients with ischemic MMD and eight patients with hemorrhagic MMD completed the TMT-A and TMT-B. The FA values of right AF were significantly correlated with working memory (*r* = 0.58, *p* = 0.010) and TMT-A direct scores (*r* = 0.59, *p* = 0.026) (Figures 5C,D). A correlation trend was found between the FA values of right AF and MMSE, as well as MoCA and TMT-B (Figures 5A,B,E), except TMT B-A (Figure 5F).

## Discussion

In this study, we revealed that the cortical thickness of the right MTG of hemorrhagic MMD was significantly greater than both ischemic MMD and the HC group. In addition, the mean FA values of right AF, which connects the MTG to frontal gyrus (22), were significantly higher in the hemorrhagic MMD group than both ischemic MMD and HCs, suggesting the potentially increased integrity of fibers starting from/ending to right MTG. More interestingly, the microstructural property of right AF was significantly associated with the cortical thickness of right MTG and also linked to the cognitive performance among patients with MMD.

In the most of previous research on ischemic MMD or mixed MMD groups, gray matter atrophy or cell density decrease was generally found (6, 7, 24). This study provides the first evidence of cortical thickness increase in hemorrhagic MMD, and the increased cortical thickness mainly happens on the right MTG. Cortical thickness reflects the overall content of neurons, the cellular composition, and the cytoarchitectonic organization of the specific cortical region (25). Therefore, MRI-based cortical thickness estimation is often used to observe the integrity of cerebral cortex on the microstructural level (26). Fierstra et al. (27) raised several possible factors affecting cortical thickness: loss and proliferation of neuroglial tissue; cortical blood flow; extracellular matrix content; and changes in the content of the myelin sheath in the cortex. In this study, we found that the cortical thickness in MMD is positively correlated with the cortical CBF and its associated fibers' FA, suggesting that the increased cortical blood flow and the increased myelin sheath or axon density of connected fibers might be the underlying mechanism of the observed cortical thickness increase in hemorrhagic MMD, although more studies are still needed to validate this hypothesis.

Regional changes of the cortical structure of a specific brain region are also likely to reflect connectivity changes crossing the region (18, 28, 29). Therefore, we further investigated changes in the white matter tracts associated with the thickened right MTG. Since SLF and AF were the most important association fiber tracts communicating with the temporal and frontal lobes, which especially had projections to the MTG (22, 23), we further explored the integrity of two aforementioned white matter tracts. The results showed that the FA values of right AF in hemorrhagic MMD were significantly higher than ischemic MMD and the HC group. The AF plays a key role in the right hemisphere in visuospatial processing and some aspects of language processing (30). Meanwhile, visual search, perceptual speed, processing speed, working memory, and general intelligence are among the most frequently cited constructs thought to contribute to TMT performance. Especially, TMT-A requires mainly visuoperceptual abilities (15). The present study confirmed this relationship between the FA values of right AF and visuospatial processing ability. The results suggested that the integrity of right AF was associated with variable cognitive functions among patients with MMD, especially in working memory and visuospatial processing ability.

The increased cortical thickness of MTG possibly results from the collateral circulation of both anterior choroidal arteries (AChA) and posterior choroidal arteries (PChA) or posterior cerebral arteries (PCA). Angiography confirmed that the AChA and PChA significantly developed and extended in hemorrhagic hemispheres than in ischemic hemispheres (31). Meanwhile, a recent cross-sectional study, exploring the cortical distribution of fragile dilated periventricular arteries, found the outflow of choroidal arteries to the cortex *via* medullary arteries in hemorrhagic MMD (32). It proved that the choroidal arteries anastomose reaching the cerebral cortex. The laterality of cortical thickness difference might be affected by the laterality of hemorrhage in the right hemisphere. There was a significant difference between ischemic and hemorrhagic MMD in the percentage of patients mainly affected in the right hemisphere, 12.5 and 66.7%, respectively. These results were consistent with previous studies which demonstrated that the dilated AChA or the abundant collateral circulation from PCA were related to the hemorrhagic events (33, 34). Increased cortical thickness were found in other brain regions in hemorrhagic MMD, such as the SMG and IFG, but there was no statistical difference between groups when using a strict statistical analysis. MTG may not be the only special gyrus; thus, increased cortical thickness in the parts of one hemisphere might be the radiological indicator to efficiently predict the occurrence of hemorrhagic stroke or hemorrhagic transformation in MMD. In addition, the tailored targeting bypass strategy, which was a novel technical modification, was promoted to prevent hemorrhage in MMD in recent years. It illustrated an approach that the superficial temporal artery anastomosed with targeted cortical vessels distributing from choroidal arteries (35). Therefore, the region of MTG around Sylvian fissure might be the potential anastomotic site in hemorrhagic MMD to alleviate the pressure of fragile collateral circulation and further lower its rupture rate. Future studies have to prove that if the medullary arteries are more abundant beneath or richer collateral circulation in the area of thickened cortex.

There are two reasons for choosing patients with cerebral lesions <3 mm. At first, the lesion itself might affect the cerebral cortex if the lesion is large. Thus, many neuroimaging studies of MMD have strict inclusion criteria, e.g., “no evidence of infarct in the brain” (7, 36). Primary intraventricular hemorrhage (IVH) happens 37.6% in hemorrhagic MMD, while 80.6% are intraparenchymal hemorrhages accompanied by IVH (37). We exclude patients with cortical lesions larger than 3 mm, these cortical lesions are not pure microbleeds, they can be residual minor destruction of brain structures after the absorption of hematoma. Besides, especially in patients with primary IVH, there is always no evidence of lesions in the parenchyma. Second, the large cerebral lesion could affect the imaging preprocessing as most brain structure segmentation or parcellation algorithms are typically optimized for normal or close to normal brains without major morphologic aberrations (38), apparent brain lesions would inevitably render study results inaccurately.

In addition, limitations should be noted in this study. The main limitation of this study is the small sample size. However, we have implemented strict statistical analysis to overcome this limitation. For example, we used the criterion of a cluster-wise threshold of *p* < 0.001 with the Bonferroni correction to determine statistical significance on the cortical thickness between two groups, and the ANOVA tests with Tamhane's T2 correction were performed in a *post-hoc* analysis. After performing these strict statistics, we believe that the finding is promising, and it is important to report these interesting results at first, which could help the collection of more patients with MMD to further validate the current conclusion. Second, our study excludes the patients that have apparent cerebral lesions in structural images, but it may exclude those presumably with more severe disease. So, the study results may apply to the patients with mild MMD. Third, the cortical segmentations were generated from an initial automatic segmentation with FreeSurfer's Desikan–Killiany atlas, whereas MNI152 was used for ASL-CBF images registration, so a slight difference may exist between templates. Fourth, we used the higher *b* value and increased number of diffusion directions for tractography, since they would help to better differentiate multiple fibers in tractography. However, *b* = 3,000 would reduce the image signal-to-noise ratio (SNR) dramatically, so an optimum *b* value of 2,000 s/mm^2^ would be recommended when doing tractography (39). Fifth, we were measuring the relative CBF (rCBF) rather than the absolute CBF value because of the elongated arterial transit artifacts of MMD itself. Finally, since we did not collect the cognitive assessment from HCs, comparison was made just between ischemic and hemorrhagic MMD groups.

## Conclusions

A specially brain structural feature differing hemorrhagic MMD from ischemic MMD and HCs is the increased cortical thickness on right MTG, which could potentially be induced by the increased CBF and integrity of connected fibers on this brain region. More interestingly, these brain structural features were correlated with the cognitive performance of patients, which are important to understand the clinical symptoms and pathophysiology of MMD, and further applied to clinical practice.

## Data Availability Statement

The original contributions presented in the study are included in the article/Supplementary Material, further inquiries can be directed to the corresponding author/s.

## Ethics Statement

The studies involving human participants were reviewed and approved by the Human Research Ethics Committee of Second Affiliated Hospital of Zhejiang University. The patients/participants provided their written informed consent to participate in this study.

## Author Contributions

LW and RB conceived and designed the paper. JH and YL wrote the paper and collected the data. JC, YT, and YC assisted in writing the paper. DX assisted in the MRI scan. DX and LW reviewed all imaging data. LZ performed the cognitive assessment. ZL and YZ contributed to analyze data. All authors contributed to the article and approved the submitted version.

## Funding

This study was supported by grants from the National Natural Science Foundation of China (No. 81870910) and the Natural Science Foundation of Zhejiang Province (No. Y18H090007).

## Conflict of Interest

The authors declare that the research was conducted in the absence of any commercial or financial relationships that could be construed as a potential conflict of interest.

## Publisher's Note

All claims expressed in this article are solely those of the authors and do not necessarily represent those of their affiliated organizations, or those of the publisher, the editors and the reviewers. Any product that may be evaluated in this article, or claim that may be made by its manufacturer, is not guaranteed or endorsed by the publisher.
