# Supplementary material for: Moyamoya Disease With Initial Ischemic or Hemorrhagic Attack Shows Different Brain Structural and Functional Features: A Pilot Study
Source: Front Neurol. 2022 May 13;13:871421. doi: 10.3389/fneur.2022.871421 (PMC9136066; doi:10.3389/fneur.2022.871421)
Supplement: Supplementary file 1 [file Table_1.DOCX]

**Supplementary Table**  Demographics and clinical characteristics of 22 MMD patients

| **Subject** | **Age** | **Gender** | **Clinical manifestation** | **Time to onset** | **Mainly Affected Hemisphere** | **Smoking** | **Alcohol** | **HTN** | **Diabetes** | **HPL** | **Comorbidity** | **Suzuki stage** |
| --- | --- | --- | --- | --- | --- | --- | --- | --- | --- | --- | --- | --- |
| P001 | 51 | F | Dizziness and headache | 2 years | / | N | N | N | Y | N | Gastritis | 5 |
| P002 | 30 | M | TIA | 1 month | left | Y | N | N | N | N | Erosive gastritis | 2 |
| P003 | 45 | M | TIA | 1 month | left | Y | N | Y | N | N | Chronic colonitis | 3 |
| P004 | 44 | F | IVH | 3 months | right | N | N | N | N | Y | / | 3 |
| P005 | 34 | F | IVH | 3 months | left | N | N | N | N | Y | ACoA | 1 |
| P006 | 36 | M | Thalamic hemorrhage with IVH | 3 months | right | Y | N | N | N | N | / | 2 |
| P007 | 42 | F | IVH | 3 months | right | N | N | N | N | N | / | 3 |
| P008 | 44 | F | Headache | 1 year | left | N | N | N | N | Y | / | 4 |
| P009 | 53 | F | TIA | 6 months | right | N | Y | Y | N | N | / | 4 |
| P010 | 58 | F | Dizziness | 6 months | / | N | N | N | Y | N | / | 2 |
| P011 | 54 | F | TIA | 2 months | left | N | N | Y | Y | N | / | 1 |
| P012 | 45 | F | SAH with IVH | 4 months | right | N | N | N | N | N | / | 2 |
| P013 | 56 | F | Cerebral ischemia | 5 months | left | N | N | N | N | N | / | 5 |
| P014 | 57 | F | Dizziness | 6 months | / | N | N | Y | Y | N | / | 1 |
| P015 | 34 | F | Basal ganglia hemorrhage | 18 months | left | N | N | N | N | N | / | 3 |
| P016 | 50 | F | Basal ganglia hemorrhage with IVH | 6 months | left | N | N | N | N | N | / | 4 |
| P017 | 52 | F | Thalamic hemorrhage | 5 months | right | N | N | N | Y | N | / | 2 |
| P018 | 53 | F | Dizziness | 10 years | / | N | N | Y | Y | N | / | 1 |
| P019 | 52 | F | TIA | 6 months | left | N | N | N | N | N | Cervicitis | 5 |
| P020 | 56 | F | IVH | 4 months | / | N | N | N | N | N | Spinal disc herniation | 3 |
| P021 | 31 | F | TIA | 2 months | left | N | N | N | N | N | / | 2 |
| P022 | 53 | F | IVH | 3 months | right | N | N | N | N | N | Lung nodules | 2 |

Note. HTN = hypertension; HPL = hyperlipidemia; mRS = modified Rankin Scale; TIA = transient ischemic attacks; IVH = intraventricular hemorrhage; ACoA = anterior communicating aneurysm.

‘/’ in Mainly Affected Hemisphere means there is no laterality of the hemorrhage and ischemia or data loss.
