# Supplementary figures and images for: Moyamoya Disease With Initial Ischemic or Hemorrhagic Attack Shows Different Brain Structural and Functional Features: A Pilot Study
Source: Front Neurol. 2022 May 13;13:871421. doi: 10.3389/fneur.2022.871421 (PMC9136066; doi:10.3389/fneur.2022.871421)

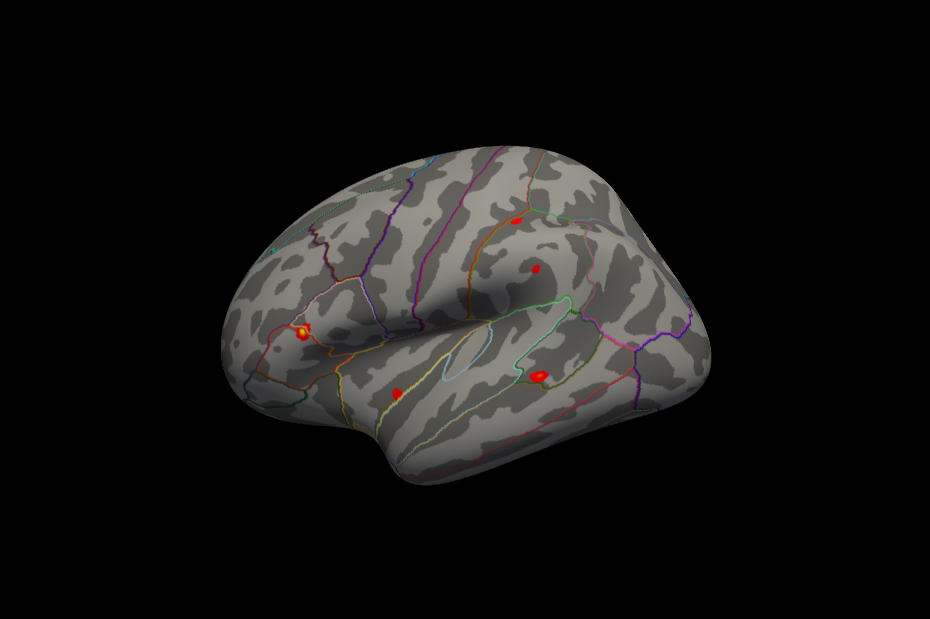

Supplement: Supplementary file 2 [file Data_Sheet_1.ZIP › hemorrhage/hemo_lh1.png]

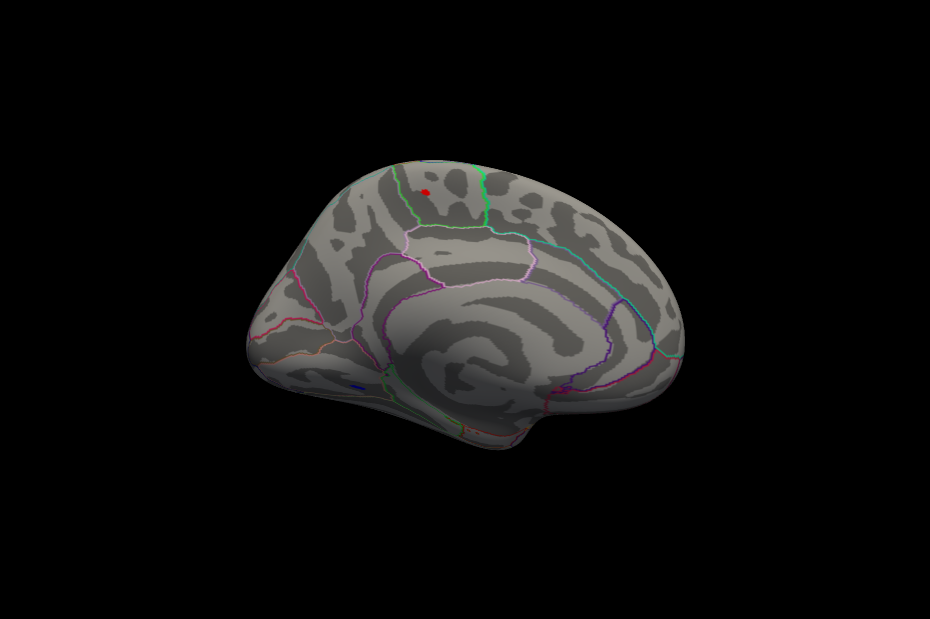

Supplement: Supplementary file 2 [file Data_Sheet_1.ZIP › hemorrhage/hemo_lh2.png]

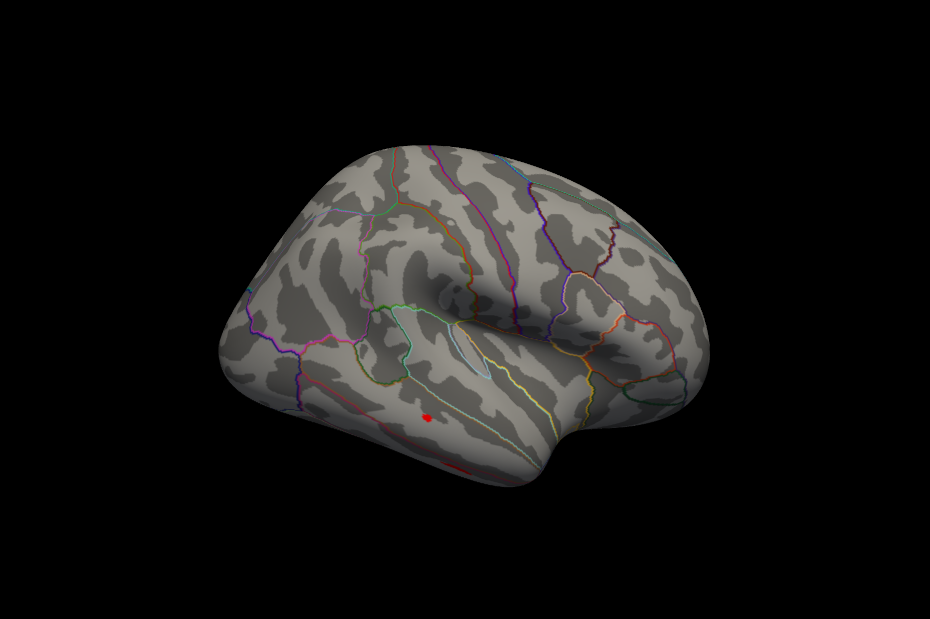

Supplement: Supplementary file 2 [file Data_Sheet_1.ZIP › hemorrhage/hemo_rh1.png]

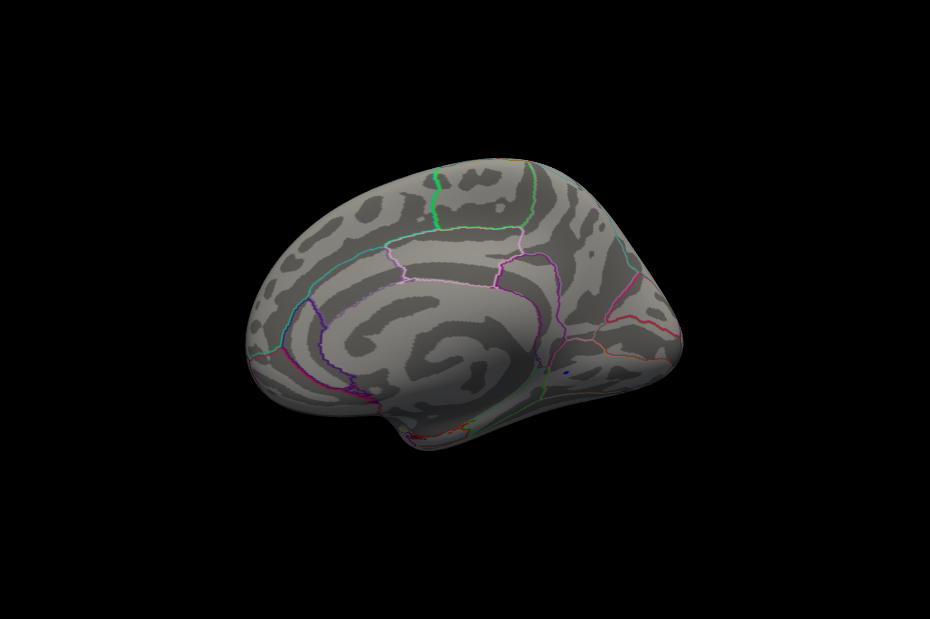

Supplement: Supplementary file 2 [file Data_Sheet_1.ZIP › hemorrhage/hemo_rh2.png]

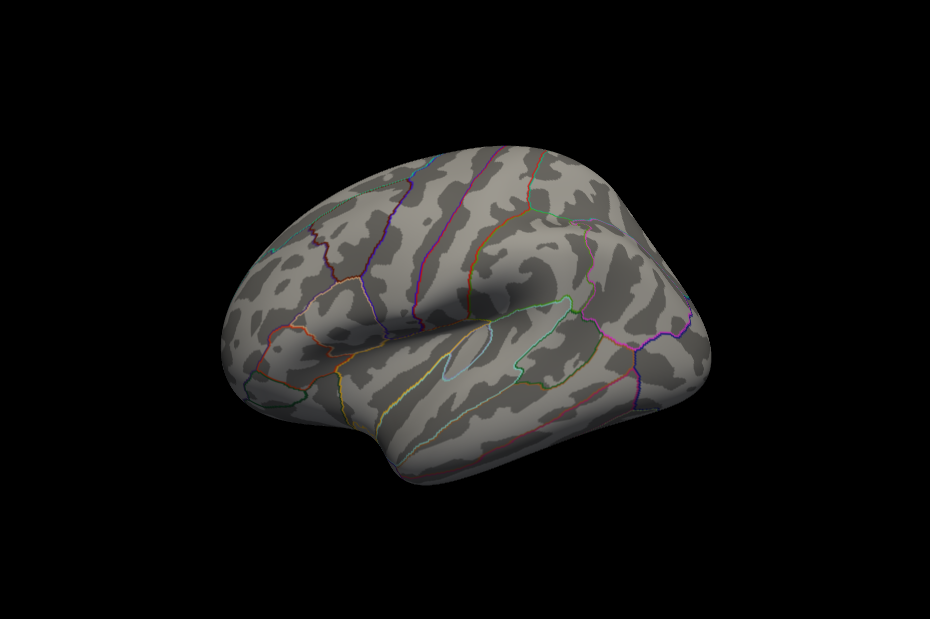

Supplement: Supplementary file 2 [file Data_Sheet_1.ZIP › ischemia/isc_lh1.png]

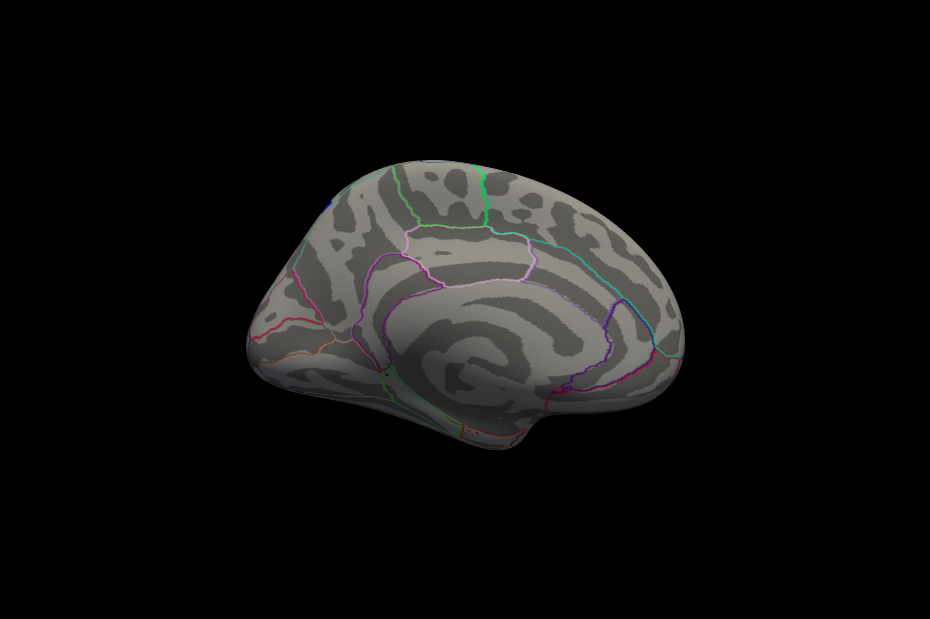

Supplement: Supplementary file 2 [file Data_Sheet_1.ZIP › ischemia/isc_lh2.png]

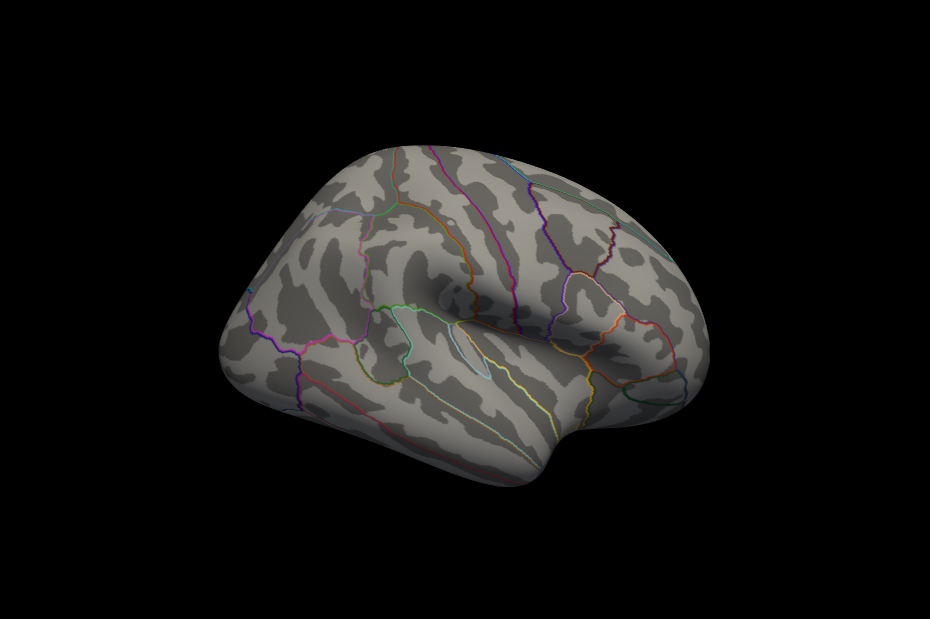

Supplement: Supplementary file 2 [file Data_Sheet_1.ZIP › ischemia/isc_rh1.png]

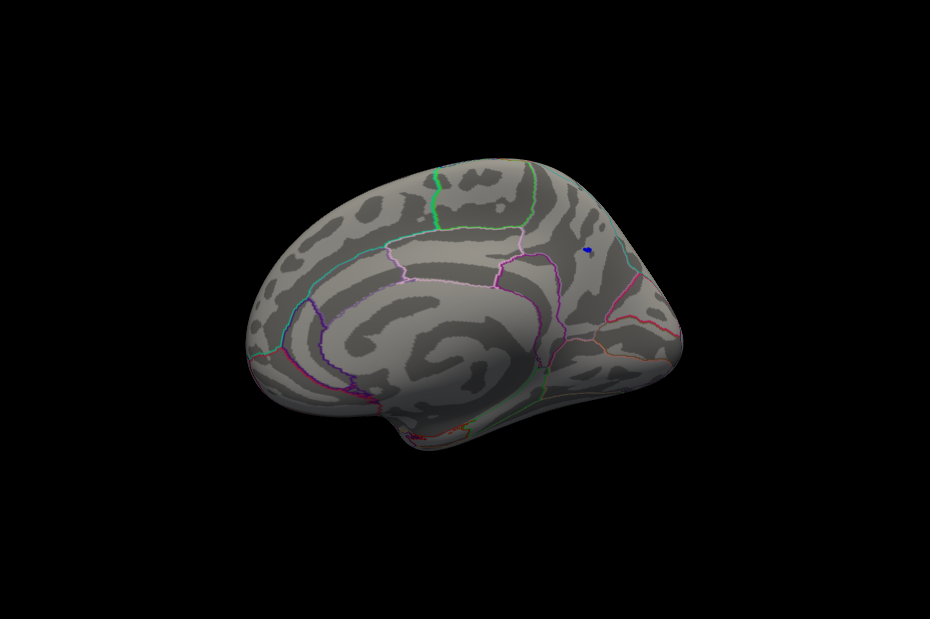

Supplement: Supplementary file 2 [file Data_Sheet_1.ZIP › ischemia/isc_rh2.png]
